# Fabrication of MEMS Directional Acoustic Sensors for Underwater Operation

**DOI:** 10.3390/s20051245

**Published:** 2020-02-25

**Authors:** Alberto Espinoza, Fabio Alves, Renato Rabelo, German Da Re, Gamani Karunasiri

**Affiliations:** Department of Physics, Naval Postgraduate School, Monterey, CA 93943, USA

**Keywords:** MEMS, directional, underwater, sensor

## Abstract

In this work, microelectromechanical systems (MEMS)-based directional acoustic sensors operating in an underwater environment are explored. The studied sensors consist of a free-standing single wing or two wings pivoted to a substrate. The sensors operate in a narrow frequency band determined by the resonant frequency of the mechanical structure. The electronic readout of the mechanical response is obtained using interdigitated comb finger capacitors attached to the wings. The characteristics of MEMS sensors immersed in silicone oil are simulated using finite element modeling. The performance of the sensors is evaluated both in air and underwater. For underwater testing and operation, the sensors are packaged in a housing containing silicone oil, which was specially developed to present near unity acoustic transmission. The measurements show that the resonant frequency of the sensors obtained in air shifts to a lower frequency when immersed in silicone oil, which is primarily due to the mass loading of the liquid. The peak sensitivity of the MEMS sensors is approximately 6 mV/Pa or −165 dB re 1 V/μPa, and the directional response shows a dipole pattern. The signal-to-noise ratio was found to be about 200 or 23 dB at 1 Pa incident sound pressure. The results show the potential of MEMS sensors to be used in underwater applications for sound source localization.

## 1. Introduction

The bearing of underwater sound sources is typically obtained using a linear array of omnidirectional hydrophones spaced proportionally to the wavelength of the source to be located [1]. These arrays require time delay, amplitude difference, or phase-weighting algorithms to determine the direction of the detected sound [2]. These sensors have evolved over the years, from relatively heavy and complex systems that required significant space onboard ships to thin light linear arrays easily handled by relatively small autonomous platforms [3,4]. An alternative approach is the use of vector sensors, which are designed to acquire vector quantities associated with the sound field [5,6,7,8,9,10,11]. The most common method to determine the direction of sound is the measurement of pressure gradient [8] or particle velocity due to the volumetric motion of the medium [9]. These variables carry the directional information of the acoustic energy propagation, which helps to identify the direction of the source. Multiple other techniques have been studied and combined to produce a directional response from underwater acoustic sensors. These include a combination of omnidirectional hydrophones to measure the pressure and an accelerometer to acquire particle velocity [10]. Commercially available vector sensors use different techniques. For example, the Microflown vector sensor measures the particle velocity by means of the temperature difference between two parallel platinum hot-wire resistors [11]. The Wilcoxon vector sensor uses three lead magnesium niobate-lead titanate (PMN-PT) crystal-based axial accelerometers and a lead zirconate titanate (PZT) omnidirectional hydrophone to extract directionality [12,13]. The measurement of particle velocity using neutrally buoyant objects that are displaced by the incident acoustic wave was also explored [14]. These sensors were constructed by mounting a velocity-sensitive device inside a rigid shell [14]. The common characteristic of these sensors is the figure eight directivity pattern.

More recently, there have been efforts to develop bio-inspired hydrophones using micromechanical structures. One of the biological systems mimicked is the lateral line tube organ of a fish. The sensor uses a pair of long cantilever beams with piezoresistors, which deform depending on the direction and pressure of the incident wave, inducing a resistance variation of the beams [15]. A bionic vector sensor was also explored using a solitary vertical cylinder that rests in the center of two crossed beams fabricated using microelectromechanical systems (MEMS) technology. Acoustic waves incident to the solitary vertical cylinder create compressive and tensile stresses in the structure. These stresses are transduced to a voltage by the piezoresistive effect of resonant tunneling diodes [16]. 

Our group is developing bio-inspired MEMS directional sound sensors that operate in air based on the hearing system of the *Ormia Ochracea* parasitic fly [17]. The main advantage of this system is the ability to determine the direction of a sound with a size much smaller than the wavelength of sound it detects. A typical sensor consists of two wings that are coupled by a bridge and attached to a substrate using two torsional legs. Sensors are built using MEMS technology on a silicon-on-insulator (SOI) substrate with integrated comb finger capacitors attached to the outer edge of the wings for electronic readout of the wings’ vibration under sound excitation [18,19]. The mechanical structure has two predominant oscillatory modes, rocking and bending, with frequencies depending on the dimensions of the structure and stiffness of the material employed. It was previously found that the bending motion of the wings has a larger amplitude and has a cosine dependence to the incident direction of sound when operated with both front and back sides exposed to sound [18]. The ability of the MEMS sensor operated in air to accurately determine the direction sound led to the investigation of the adaptability for underwater applications. In this paper, the design, fabrication and characterization of MEMS directional acoustic sensors operating in an underwater environment are described.

## 2. MEMS Underwater Sensor Design

**Figure 1:**
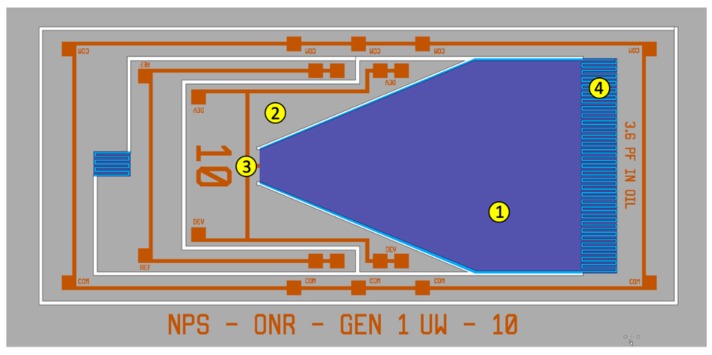
Layout diagram of the acoustic directional microelectromechanical systems (MEMS) sensor, designed for underwater operation, showing a free-standing single wing (1) connected to the substrate (2) through a pivot point (3), and the interdigitated comb finger capacitors (4) at the edge of the wing.

For underwater operation, the MEMS sensors need to be immersed in a non-conducting fluid that has acoustic impedance close to that of water. In addition, the higher sound speed in water requires the sensor dimensions to be optimized for achieving the required frequency response and directional sensitivity. The initial size of the sensor wing was chosen to be about 5 mm in length and 3 mm wide. The wing is connected to the substrate using a wedge-shaped section, as shown in Figure 1.

**Figure 2:**
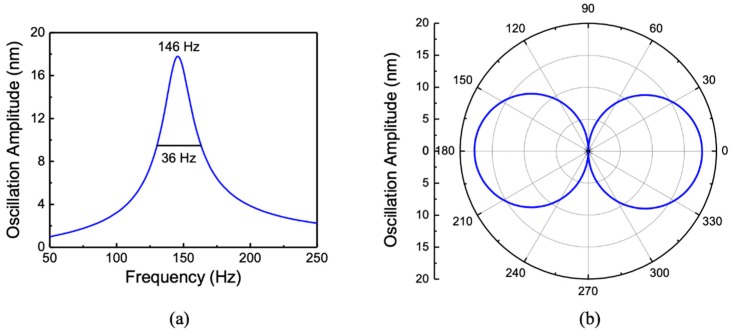
Simulated (**a**) frequency and (**b**) directional responses of the MEMS sensor when immersed in silicone oil.

The mechanical structure of the sensor is 25 μm thick and made of single crystal silicon. A comb finger capacitor that is 500 μm long and has 10 μm wide fingers with a 10 μm gap between them was placed at the edge of the wing for electronic readout of the oscillation amplitude under sound excitation. Figure 2a shows the frequency response of the sensor, obtained using COMSOL Multiphysics® finite element (FE) modeling. The simulation was performed with the sensor immersed in low-viscosity (1cSt) silicone oil. The acoustic impedance of silicone oil is close to that of the water (about 1.48 MPa·s·m^−1^). The modeling was carried out with the help of the Pressure Acoustic, Thermoviscous Acoustic, and Structural Mechanics modules of COMSOL. The incident sound wave amplitude was set to 1 Pa in the simulation. No fitting parameters were used in the simulation, and the damping was generated by interaction of the mechanical structure with the fluid. A resonant peak was found at approximately 146 Hz compared to about 880 Hz when simulated in air. Frequency reduction in oil is primarily due to the higher density (818 kg/m^3^) of oil compared to that of air. Figure 2b shows the simulated directional response (oscillation amplitude at different angles) of the sensor at 146 Hz showing the expected cosine dependence.

**Figure 3:**
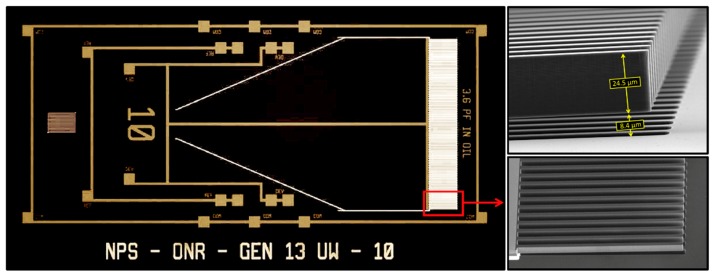
Photograph of the fabricated underwater sensor (left side) with expanded view (SEM micrograph) of the comb finger capacitors used for electronic readout of the signal. Note that the fingers from the sensor do not overlap with the fingers from the substrate due to the residual stress generated in the fabrication process.

Figure 3 shows a micrograph of the actual MEMS sensor, which was fabricated using MEMSCAP^®^ foundry service [20]. The relatively longer wing employed in the design forced us to use only a single wing to meet the MEMSCAP^®^ design rules. 

The lack of two wings eliminates the rocking mode, making the sensor to oscillate only in the bending mode. The vibrations of the wing under sound excitation are measured using comb finger capacitors attached to the edge of the wing (see the inset of Figure 3), similar to the sensors fabricated to operate in air [18]. It can be seen in the SEM image in the inset of Figure 3 that the interdigitated combs were vertically displaced without overlapping with that of the substrate. This is primarily due to the residual stress-induced tilting of the wing when released from the substrate after the fabrication. This lack of overlap reduces the overall capacitance between the wing and substrate fingers, impacting the electronic readout.

### Underwater Measurements

**Figure 4:**
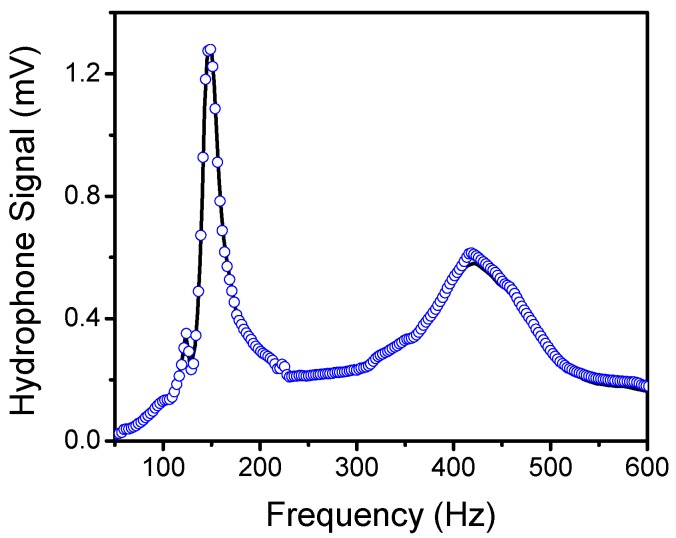
Measured frequency response of the sound projector using a calibrated hydrophone with (blue circles) and without (black line) the boot attached to it.

For underwater characterization, the sensor was immersed in non-conducting fluid with low viscosity, which was contained in a sealed housing. The 1cSt silicone oil has favorable properties for this application. A flexible boot was made of PMC-780 polyurethane attached to a flange, creating a boot structure. An electrical feedthrough was inserted into the boot to receive the signal from the sensor. First, the acoustic transmission characteristics of the boot filled with silicone oil were determined by measuring the response of a calibrated reference hydrophone (B&K 8103) inside and outside the MEMS sensor housing. Figure 4 shows the characteristic of the sound projector, which was measured using the reference hydrophone placed inside (blue circles) and outside (black line) the underwater housing. The data indicate no appreciable difference between the responses, suggesting a near unity transmission coefficient through the boot filled with silicone oil. The resonance peaks in Figure 4 are associated with the characteristics of the projector used.

**Figure 5:**
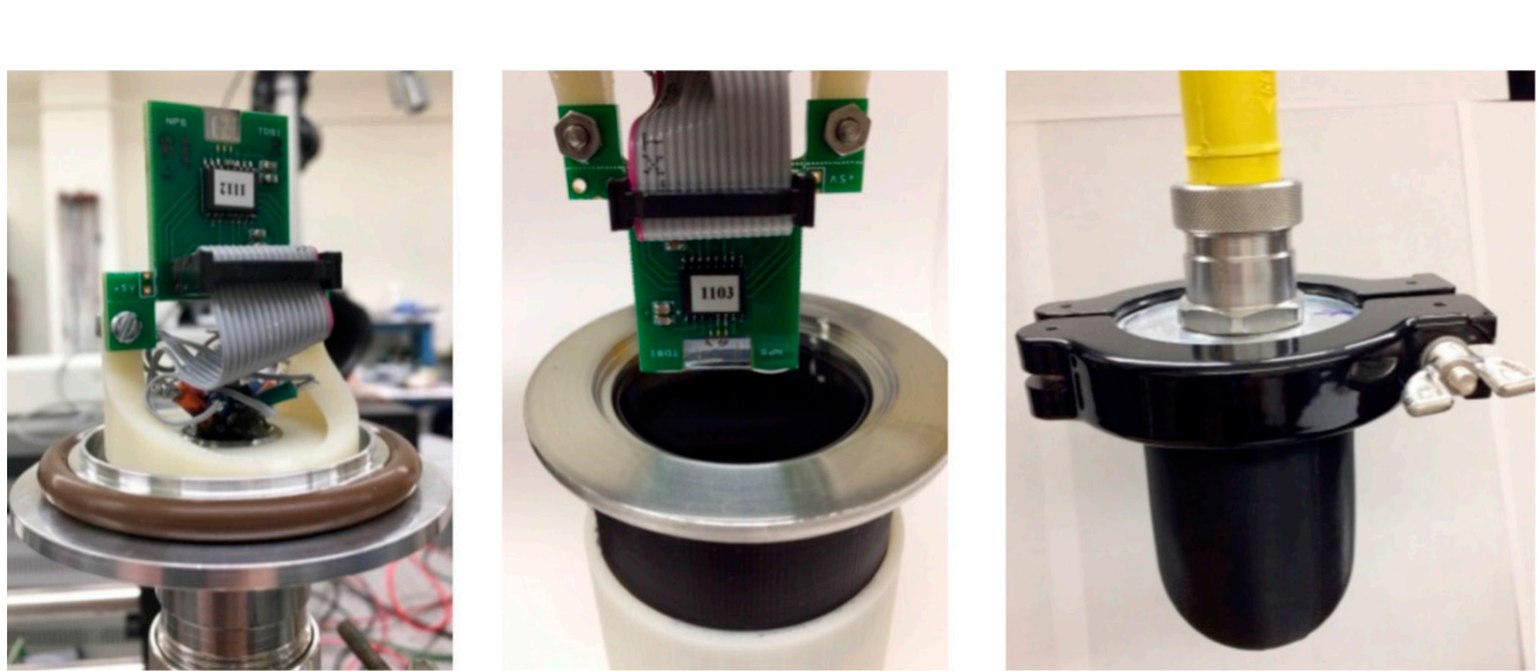
(**a**) Sensor and readout electronics mounted on a flange, (**b**) lowering sensor assembly to a housing containing silicone oil and (**c**) assembled sensor for underwater testing. The boot (black color) was made of PMC-780 polyurethane.

For underwater characterization, the sensor was mounted on a circuit board and connected to an MS 3110 capacitance to voltage converter integrated circuit. A lock-in amplifier (MLFI from Zurich Instruments) was used for recording the data. The assembled sensor with integrated readout electronics was attached to the housing and sealed in silicone oil, as shown in Figure 5. Characterization was conducted at the Acoustic Transducer Evaluation Center (TRANSDEC), which is a facility that belongs to Space and Naval Warfare Systems Command (SPAWAR). The distance between the source and sensor was determined based on the far-field distance (*d*), which can be estimated using [21]:

(1) $$d>\frac{\pi a^{2}}{\lambda }$$

where *a* is the radius of the projector (10.61 cm) and *λ* is the wavelength of the highest frequency used in the measurement. The estimated far-field distance for the frequency range of interest (50–300 Hz) was about 7.5 cm.

**Figure 6:**
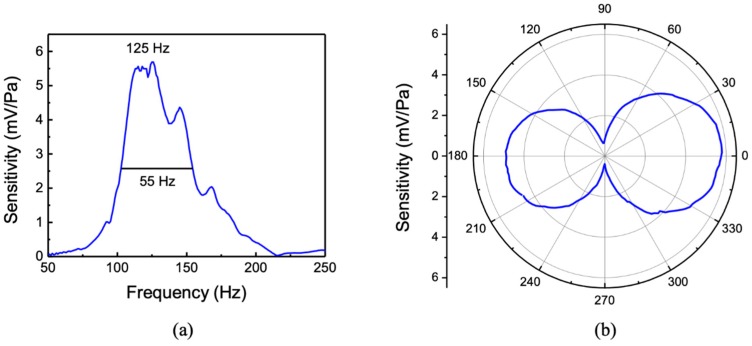
(**a**) Frequency responses and (**b**) directional response operating in an underwater environment.

During the characterization, the MEMS sensor assembly was suspended in the tank about 2 m away from the sound projector at a depth of about 6 m. A calibrated hydrophone was co-located with the sensor to provide the sound pressure. The measured sensitivity (mV/Pa) of the sensor over the frequency range of 50–250 Hz is shown in Figure 6a. The peak sensitivity was found to be about 5.5 mV/Pa or −165 dB re 1 V/μPa. The measured resonant peak position is about 20% lower than that of the simulation shown in Figure 2a. The measured full width at half maximum (FWHM) was about 55 Hz compared to about 40 Hz obtained from the simulation without using any adjustable parameters. Both the drag from the wings and fluid flow between comb fingers (Couette flow) contribute to damping [22]. The directional response of the sensor was measured at the peak frequency (125 Hz) and is shown in the polar plot in Figure 6b. The directional response showed a cosine dependence and agrees well with the predicted response in Figure 2b. The cosine directional pattern is originated by the interaction of sound from the top and bottom sides of the wings, making it act as a pressure-gradient microphone [18]. The slight asymmetry of the measured pattern is most likely to be due to the effect of the housing flanges and clamps.

**Figure 7:**
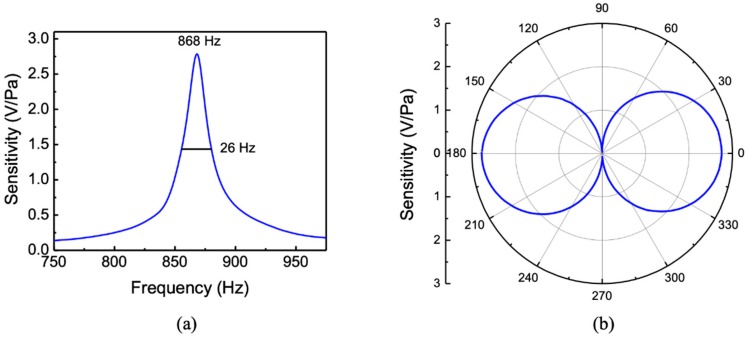
Measured (**a**) frequency response and (**b**) directional response of the sensor in air. The directional response was measured at the peak frequency of 868 Hz.

For comparison with the underwater measurements, the characteristics of the sensor were also measured in air in an anechoic chamber. Figure 7a shows the measured sensitivity of the sensor in air, showing the bending resonance at around 868 Hz. The sound pressure at the sensor location was measured using a calibrated microphone. The resonance occurred at a higher frequency (868 Hz) in air than the measured frequency in silicone oil (125 Hz) due to the lack of mass loading. The peak sensitivity was found to be about 2.7 V/Pa. Measurement of the directional response of the sensor in air was also performed at resonance (868 Hz) and is shown in Figure 7b, giving a cosine directional pattern as expected. 

## 3. Dual-Wing Sensor Design

**Figure 8:**
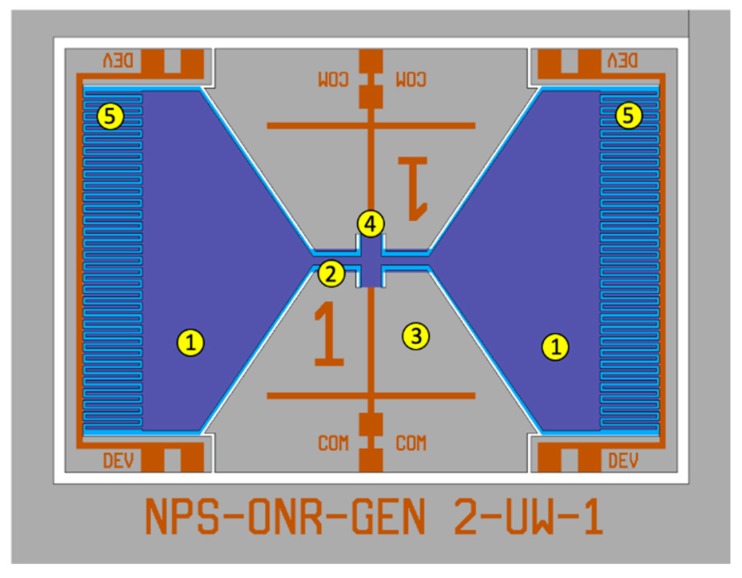
Layout diagram of the acoustic directional MEMS showing two free-standing wings (1) interconnected by a bridge (2), anchored to the substrate (3) by a torsional leg (4), and the interdigitated comb finger capacitors (5) at the edge of the wings.

One of the drawbacks of the one-wing design is the lack of overlap between the fixed and moving comb fingers due to the use of a relatively long wing. The longer wing provides a larger oscillation amplitude, which can generate a stronger electrical signal; however, the lack of overlap between the fingers reduces the amount of capacitance change under sound excitation, resulting in a smaller electronic signal [19]. One way to make the fingers overlap is to reduce the length of the wing; however, this causes an undesirable reduction in the oscillation amplitude. This can be compensated by adding a second wing, as demonstrated in our earlier MEMS sensors operated in air [23]. Figure 8 shows the schematics of a two-wing MEMS sensor design for operation underwater. The length of the wing was reduced from 5 to 2.5 mm, and the gap between fingers was also reduced from 10 to 5 μm. The reduction of the gap will further enhance the mechanical to electrical transduction due to increased capacitance. Note that the reduced gap increases the component of damping generated by these structures, which in turn could reduce the oscillation amplitude. Nevertheless, it was found in the simulation that the main contribution to damping comes from the drag, which depends on the area of the wings.

**Figure 9:**
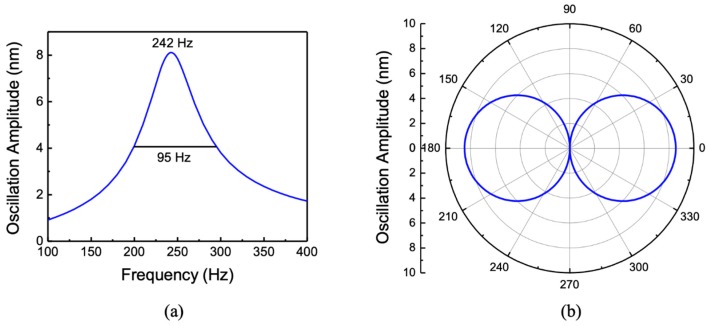
Simulated (**a**) frequency response and (**b**) directional response of the MEMS acoustic sensor in silicone oil. The directional response was simulated at 242 Hz (bending resonant frequency).

As before, the simulation was performed by immersing the sensor in silicone oil. Figure 9 shows the simulated frequency and directional responses. The incident sound wave amplitude was kept at 1 Pa. The bending resonance peak of the sensor was found to be around 242 Hz, and the simulated directional response at resonance showed the expected cosine behavior. The FWHM was about 95 Hz, which is higher than that obtained for the single-wing configuration. This may be due to the use of a smaller gap between the comb fingers in the two-wing configuration, since the total area of the wings is close to that of the single-wing design.

**Figure 10:**
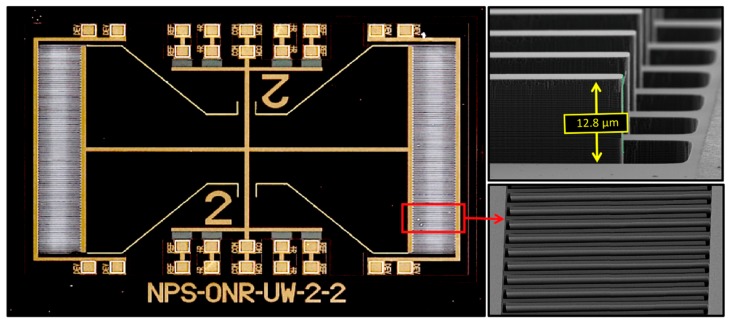
Photograph of the fabricated two-wing sensor (left side) with expanded view (SEM micrograph) of the comb finger capacitors used for electronic readout of the signal. Note that the fingers from the sensor overlap with the fingers from the substrate due to the reduced size of the wing.

Figure 10 shows a fabricated sensor using a two-wing configuration. The SEM images in Figure 10 clearly show that the combs are overlapped, which should provide higher electronic sensitivity [19]. The sensor was mounted on a custom-fabricated circuit board with an MS3110 capacitive readout integrated circuit, which is similar to the single wing configuration. The comb finger capacitors from both wings were connected in parallel in order to increase the electronic response.

### 3.1. Underwater Measurements

**Figure 11:**
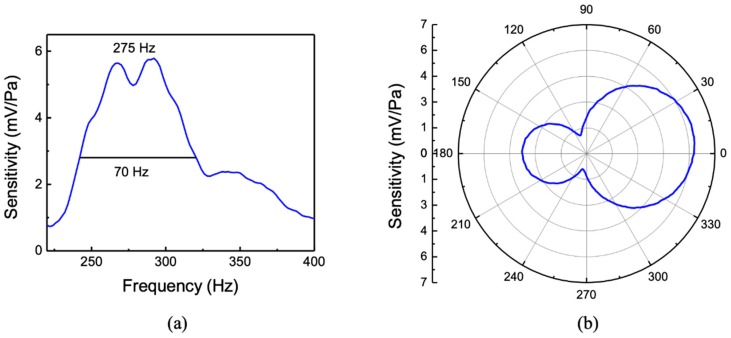
(**a**) Measured sensitivity with frequency and (**b**) measured directivity pattern at 275 Hz.

During underwater testing, frequency responses of the MEMS sensor and a reference hydrophone were measured simultaneously from 220 to 400 Hz by placing them at equal distances from the sound projector. The measured sensitivity (mV/Pa) of the sensor is shown in Figure 11a. A relatively broad resonant peak centered around 275 Hz can be observed with a maximum sensitivity of about 6 mV/Pa or −165 dB re 1V/µPa. The increased bandwidth, compared to that of the single-wing sensor, arises from the additional damping generated by the second wing with the combs as well as a smaller gap between the comb fingers. Note that in spite of the broader response of this sensor, the sensitivity at the resonant peak is similar to that measured for the single-wing sensor. This can be explained by the increased electrical signal generated by the overlap of comb fingers. The simulated resonant peak shown in Figure 9a is found to be about 10% below that of the measured.

The directional response of the MEMS sensor was measured by rotating it at the peak frequency of 275 Hz. The directivity pattern at 275 Hz is shown in Figure 11b, giving an asymmetric figure eight directivity pattern. Again, the asymmetric response is most likely to be due to the housing flanges and clamps that have asymmetric configurations. Further measurements are needed to fully understand the origin of the non-zero minima observed in Figure 11b. 

**Figure 12:**
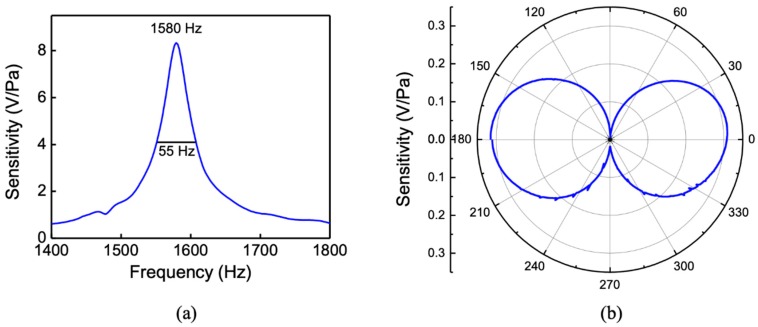
Measured (**a**) sensitivity and (**b**) directivity pattern of the sensor in air. The directivity pattern was measured at 1580 Hz.

The sensor was also characterized in air for comparison with the underwater measurements. Figure 12a shows the measured frequency response in air showing a resonant peak at 1580 Hz due to the bending motion of the two wings. 

The higher resonant frequency compared to the single-wing design is due to the smaller wing size, which has a lower mass and hence an increased resonant frequency. As expected, the peak sensitivity increased to more than twice that of the single-wing sensor (about 8 V/Pa) due to an increase in capacitance with overlapped combs and a smaller gap between them as well as the addition of the second wing to compensate for the reduced length. A polar plot of the measured directivity pattern at the resonant peak (1580 Hz) is shown in Figure 12b. The maximum amplitude is at normal incidence, while the null occurs when the sound wave travels parallel to the wings of the sensor similar to a pressure gradient microphone [18].

### 3.2. Noise Measurement

**Figure 13:**
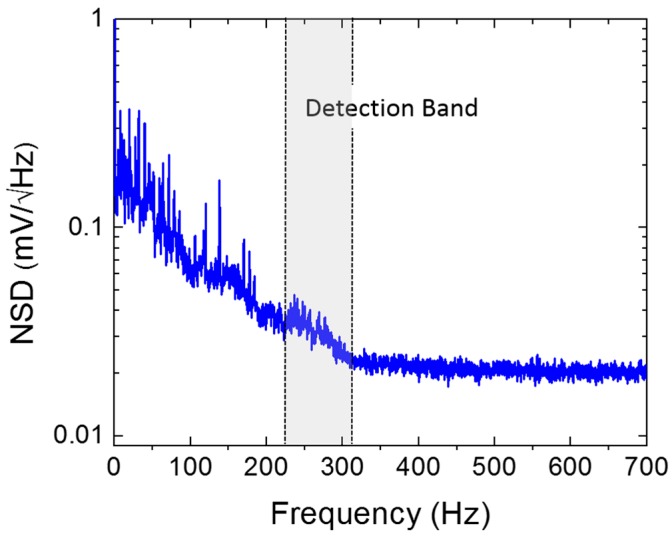
Measured noise spectral density of the two-wing sensor. The measurement was performed over a 6.5 kHz band inside an anechoic chamber with a noise floor of approximately 28 dB. The sensor readout electronics were programed with the same parameters used to obtain the results shown in Figure 12.

The noise spectrum of the two-wing sensor was measured in an anechoic environment, using the lock-in amplifier over a band of 6.5 kHz. The sensor readout electronics were programed with the same parameters used to obtain the data shown in Figure 11. The measurement reference frequency was set to 8 kHz, and the frequency span was set to 16 kHz. A fast Fourier transform (FFT) based on the input data displays only half of the frequency span (up to the Nyquist frequency). In addition, an optimal alias rejection processing is used, resulting from the selected detection bandwidth and signal sampling rate. Each scan was acquired with approximately 0.2 Hz frequency resolution. Figure 13 shows the measured noise spectral density (V/√Hz) after averaging 100 times in the frequency range of interest, from 0 to 700 Hz. Outside this range, the noise spectral density was predominantly flat, presenting a white-noise characteristic. The dominant source of noise may be attributed to the readout electronics except in the frequencies between 100 and 300 Hz, in which it is most likely due to a combination of the 1/f and mechanical vibration of the sensor at the rocking and bending modes. The signal-to-noise ratio was estimated by assuming that the sensor is operated near the resonance with a 70 Hz bandwidth (see Figure 12). For a sound pressure level of 1 Pa, the average signal power in this band is about 22.5 × 10^−6^ (V^2^). The noise power was obtained, integrating the square of the noise spectral density (NSD) over the same band (see Figure 13) and is about 112 × 10^−9^ (V^2^), giving a signal-to-noise ratio of about 200 or 23 dB.

## 4. Conclusions

MEMS-based directional sound sensors with two different configurations were studied for potential application in an underwater environment. Two sensors were designed using finite element modeling (COMSOL Multiphysics^®^), and their frequency and directional responses in air and immersed silicone oil surrounded by water were simulated. The sensors were fabricated using the MEMS commercial foundry MEMSCAP^®^ and fully characterized in air. A close agreement between prediction and measurement in an anechoic chamber was obtained. A housing was designed for testing the sensor in an underwater environment, and the materials employed were found to exhibit nearly 100% acoustic transmission. The measured resonant peaks of the two sensors were close to those of the simulations, while the directional responses showed the expected dipole behavior associated with pressure gradient microphones. It was found that a greater overlap of combs could be achieved by reducing the length of the wing without affecting the overall sensitivity. The noise measurement of the sensor with readout electronics gave a signal-to-noise ratio of about 23 dB at 1 Pa incident sound pressure. These preliminary results indicate that our MEMS directional acoustic sensors have the potential to be used for underwater applications, especially in resonant mode, which can be tuned by design.
